# The Use of Three-Dimensional DNA Fluorescent In Situ Hybridization (3D DNA FISH) for the Detection of Anaplastic Lymphoma Kinase (ALK) in Non-Small Cell Lung Cancer (NSCLC) Circulating Tumor Cells

**DOI:** 10.3390/cells9061465

**Published:** 2020-06-15

**Authors:** Arutha Kulasinghe, Yenkai Lim, Joanna Kapeleris, Majid Warkiani, Ken O’Byrne, Chamindie Punyadeera

**Affiliations:** 1 The School of Biomedical Sciences, Institute of Health and Biomedical Innovation, Queensland University of Technology, Kelvin Grove, QLD 4035, Australia; y.lim2@uq.edu.au (Y.L.); joanna.kapeleris@qut.edu.au (J.K.); k.obyrne@qut.edu.au (K.O.); chamindie.punyadeera@qut.edu.au (C.P.); 2 Saliva and Liquid Biopsy Translational Research Team, Institute of Health and Biomedical Innovation (IHBI), Queensland University of Technology (QUT), Kelvin Grove, QLD 4035, Australia; 3 Translational Research Institute, Woolloongabba, QLD 4102 Australia; 4 The School of Biomedical Engineering, University of Technology Sydney, Ultimo, NSW 2007, Australia; Majid.Warkiani@uts.edu.au; 5 Princess Alexandra Hospital, Woolloongabba, QLD 4102, Australia

**Keywords:** circulating tumor cells, anaplastic lymphoma kinase, non-small cell lung cancer

## Abstract

Tumor tissue biopsy is often limited for non-small cell lung cancer (NSCLC) patients and alternative sources of tumoral information are desirable to determine molecular alterations such as anaplastic lymphoma kinase (ALK) rearrangements. Circulating tumor cells (CTCs) are an appealing component of liquid biopsies, which can be sampled serially over the course of treatment. In this study, we enrolled a cohort of ALK-positive (n = 8) and ALK-negative (n = 12) NSCLC patients, enriched for CTCs using spiral microfluidic technology and performed DNA fluorescent in situ hybridization (FISH) for ALK. CTCs were identified in 12/20 NSCLC patients ranging from 1 to 26 CTCs/7.5 mL blood. Our study revealed that 3D imaging of CTCs for ALK translocations captured a well-defined separation of 3′ and 5′ signals indicative of ALK translocations and overlapping 3′/5′ signal was easily resolved by imaging through the nuclear volume. This study provides proof-of-principle for the use of 3D DNA FISH in the determination of CTC ALK translocations in NSCLC.

## 1. Introduction

Lung cancers are the leading cause of cancer-related deaths in the world with 85% of all cases being non-small cell lung cancer (NSCLC). Patients are often diagnosed at an advanced stage, where the immediate prognosis is often poor, resulting in poor five-year survival rates of 23% [1]. A subset of patients, approximately 3–7% of NSCLC, present with anaplastic lymphoma kinase (ALK) rearrangements. These are usually associated with never smokers representing a younger patient age group with signer or acinar histology. In tumor biopsy, ALK is determined to be positive when >50% of counted nuclei (typically out of 50) are positive. If <10% are positive, it is considered negative and equivocal when this ranges between 10% and 50% positivity where an additional 50 nuclei are analyzed and scored out of 100 total nuclei to determine the ALK score [2,3]. These patients are eligible for ALK-targeted therapies such as crizotinib (tyrosine kinase inhibitor). Crizotinib was originally developed as an inhibitor of mesenchymal–epithelial transition factor (c-MET) and found to be a potent inhibitor of ALK [4]. Patients treated with crizotinib have better response rates and longer progression-free survival compared with standard chemotherapy, however, patients often undergo relapse due to the emergence of resistance mechanisms [5]. To better understand the emergence of treatment resistance, it is crucial to be able to sample the tumor over the course of therapy [6]. In the case of lung cancer, this can be invasive and oftentimes challenging due to the location of the tumor. Therefore, minimally invasive means of sampling and capturing the tumor activities in real time are needed [7,8,9].

Liquid biopsies have emerged as an alternative source of tumor material that can be minimally invasively and serially sampled over the course of therapy in NSCLC [10,11]. In recent years, EML4-ALK rearrangements have been reported to be found in circulating tumor cells (CTCs) [12,13,14,15,16,17]. Fluorescent in situ hybridization (FISH) allows for the visualization of the organization and positioning of chromosomes and subchromosomal regions. 3D FISH provides spatial organization of the chromosomes within the nuclei, where gene-to-gene distances can be assessed with more precision than the classical 2D FISH [18]. However, 3D FISH experimentation has the additional steps involved such as region of interest selection, cell segmentation, detection of signal within the nuclear space, and localization of genes [19,20]. In this proof-of-principle study, we sought to investigate the role of ALK translocations from a cohort of ALK-positive and ALK-negative NSCLC patients.

## 2. Results

**Figure 1:**
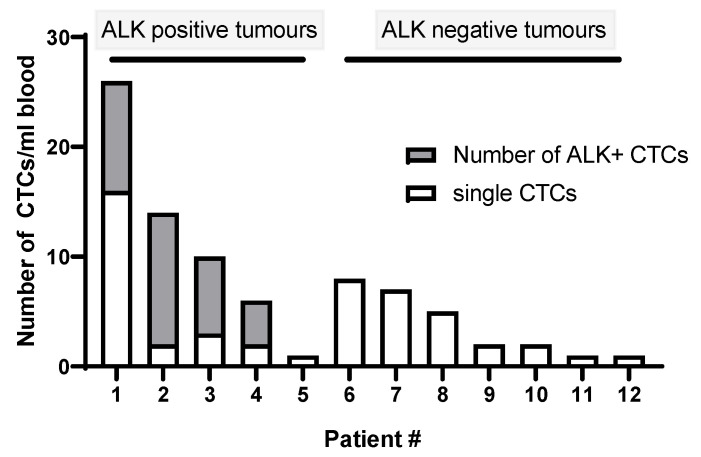
Of the 8 patients with ALK-translocated tumors, 5 had detectable CTCs (Patients # 1–5); 4/5 of the CTC-positive patients had ALK-rearranged CTCs, the percentage of which is shown in the grey. In ALK-negative tumors, CTCs were detected in 7/12 cases (Patients # 6–12).

**Figure 2:**
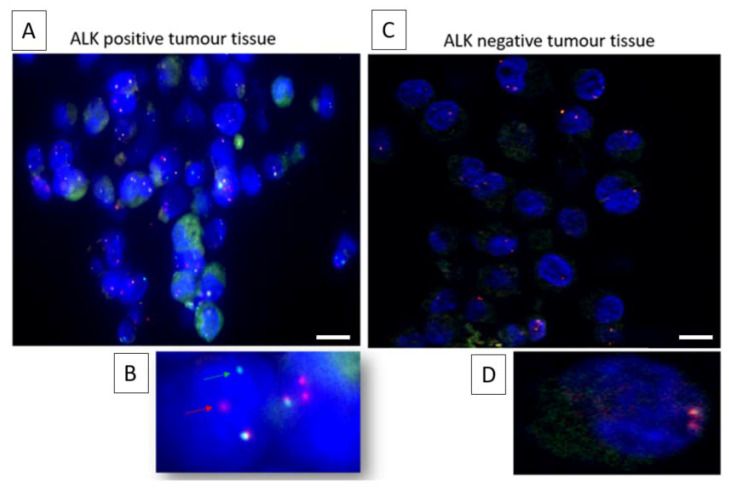
2D ALK DNA FISH performed on positive and negative control tumor tissue slides. (**A**) Low magnification ALK-positive tumors show the (**B**) characteristic split of the 3′ (red) and 5′ (green) signals at higher magnification whereas (**C**) ALK-negative tumors show the native phenotype with the (**D**) absence of an ALK translocation. Multiple fused signals (overlapping/adjacent) are observed in the nucleus of the ALK-negative tumor. Figure legend represents 10 µm.

**Figure 3:**
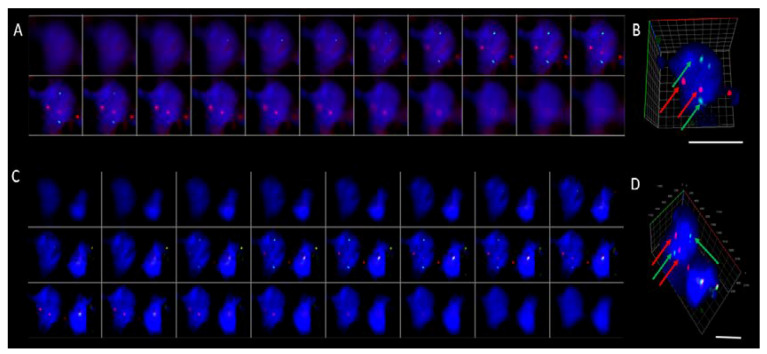
Examples of ALK-translocated CTCs imaged using 3D DNA FISH. (**A**,**C**) The gallery of sequential z-stacks showing the additional signals found within the nuclear volume. (**B**,**D**) The maximal orthogonal view showing the 3D composite image of the CTC. The 3′-ALK (red), 5′-ALK (green), and DAPI (blue). Scale bar represents 10 µm. Video of imaging through the nuclear volume (Supplementary Materials).

For this study, a cohort of ALK-positive (n = 8) and ALK-negative (n = 12) NSCLC patients were recruited. ALK for tumor tissue was confirmed by ALK protein staining (ALK CDx assay) using the Ventana ALK (C5F3) companion diagnostic test (Roche Diagnostics, Indianapolis, IN, USA). All ALK-positive NSCLC patients received a form of tyrosine kinase inhibitor (crizotinib, ceritinib, or alectinib). CTCs were detected in n = 13/20 patient samples (5/8 ALK-positive cases and 7/12 ALK-negative cases) ranging from 1 to 26 CTCs/7.5 mL blood (Figure 1). No CTC clusters were found in the 12 CTC-positive samples. Prior to staining the CTC-positive slides, control tissue was assessed to determine the staining patterns of the Vysis LSI ALK break-apart probes (Abbott, Chicago, IL, USA) (Figure 2). The Vysis LSI ALK break-apart probes showed clear ALK translocations in the ALK-positive tumor slides (Figure 2A,B) and the non-ALK-translocated tumor tissue showed no signal separation (Figure 2C,D). The CTC-positive cases were further analyzed using 3D ALK-DNA FISH (Figure 3A,C) individual images whilst imaging through the nuclear volume of CTCs (Figure 3B,D) the maximal orthogonal view showing the 3D composite image of the CTC. Z-stacks were created per CTC, which were spatially rotated to capture signals within the nuclear space and to represent the maximal orthogonal view. Deconvolution algorithms were used to further interrogate the images (constrained iterative algorithm, Zeiss). Video imaging through the nuclear volume of the CTCs showing the variation of signal within the planes is in the Supplementary Materials. Signals were validated by an experienced cytogeneticist. No ALK-translocated CTCs were found in patients with non-ALK-rearranged tumors.

## 3. Discussion

Advances in therapies targeting signaling pathways such as EGFR and ALK have led to improvements in patient survival and a reduction in toxicities in NSCLC patients. Whilst tumor tissue biopsy is desirable to make these assessments in NSCLC patients, there are limitations on the quantity and quality of tumor material available from a tissue biopsy. CTCs represent an alternative source of tumor material, to assess the landscape of primary and metastatic tumor deposits in the body by sampling blood. This represents a real-time, minimally invasive method of sampling which can be performed serially over the course of treatment to understand the dynamic tumoral changes in response to the selective pressures of treatment. A number of studies have reported on the presence of ALK-translocated CTCs in blood taken from NSCLC patients [12,14,15,17,21,22,23] with recent findings from Pailler et al., 2019, sequencing single CTCs that revealed the heterogeneity and resistance mutations in ALK-rearranged patients. The study by Pailler et al., 2019, demonstrated that various genes involved in the RTK-KRAS and TP53 pathways were found in patients with crizotinib resistance [24]. Therefore, the ability to assess the mutational changes in real time and over the course of therapy is of critical importance. The analysis of CTCs is gaining traction to determine molecular alterations which would make the patients eligible for targeted therapies, often when tumor tissue is a limiting factor to determine tumoral information [15,25]. Whilst the immediate application was developed in NSCLC, this can be extended to other molecular alterations across a number of tumor types [21].

Our study demonstrated that by spiral chip enrichment, CTC ALK rearrangements could be detected routinely using DNA FISH, and that imaging CTCs in 3D presented the 3′ and 5′ signal within the nuclear volume more readily compared to conventional 2D imaging for an assessment of ALK translocations. Imaging using 3D DNA FISH offers visual and direct results where spatial assessments of the nucleus can be made [20]. On occasions where the 3′ and 5′ signals were not clear, rotation of the CTC about an axis would enable the interrogation of the signal. Imaging through the nuclear volume presented a robust method to determine whether an ALK translocation was present or whether the 3′ and 5′ signals were in close proximity/overlapping. The measurement of nuclear distances and the number of nuclei can be time-consuming, however, the addition of image automation may provide a more routine and robust process prior to clinical implementation. A similar approach was recently described by Lim et al., 2018, using subtraction enrichment and immunostaining in situ hybridization (SE-FISH) [18]. A limitation of DNA FISH includes the harsh treatments required to prepare the chromatin for the FISH probes, which can disrupt the nuclear structure. To overcome these, strategies have been developed such as freeze–thaw permeabilization of cells and denaturation using heat, prior to probe hybridization [20]. A limitation of enrichment with the spiral technology is that CTCs that are of comparable size to WBCs or smaller are often not captured using this size-based CTC capture methodology [26]. Whilst proof-of-principle, the authors envisage expanding this study to a larger validation cohort of NSCLC patients (including patients with ALK/ROS-1 alterations) and collecting follow-up samples to determine the changes in CTCs over the course of treatment.

Tumor tissue uses a threshold of 15% of ALK-translocated cells to diagnose a patient as ALK-positive. Whilst our findings are preliminary, expansion of our study may enable the development of a CTC threshold. A number of groups have attempted to develop such a threshold for NSCLC [12,27]. In the study by Pailler and colleagues, they demonstrated that four or more ALK-rearranged CTCs per mL of blood gave a sensitivity and specificity of 100% when compared to tumor tissue. Moreover, no or one ALK-rearranged CTC was found in ALK-negative patients [12]. In our study, we did not find any ALK-translocated CTCs in the ALK-negative cohort of patients. This, in part, could be due to the combination of spiral microfluidic chip enrichment, which reduces the number of contaminating white blood cells by multiple log-fold [28,29,30,31], and the comprehensive assessment of putative CTCs using 3D DNA FISH for ALK translocations. However, this would need to be tested in a larger cohort of patients to determine an ALK cut-off for CTCs predictive of ALK rearrangements present in the primary tissue.

## 4. Materials and Methods

### 4.1. Patient Cohort

Ethics approval was obtained from the Metro South Health District Human Research Ethics Committee in accordance with the National Health and Medical Research Councils guidelines (HREC/11/QPAH/331) to collect samples from the Princess Alexandra Hospital. All methodologies were performed in accordance with these ethical guidelines and regulations. This study has institutional approval from the Queensland University of Technology human ethics committee (1100001420). Following written informed consent, 10 mL of blood was collected from n = 20 NSCLC patients prior to therapy.

### 4.2. ALK CDx Assay

The companion diagnostic test (VENTANA D5G3) was used to determine ALK protein in tumor tissue samples (formalin-fixed, paraffin-embedded tissue) stained with the BenchMark XT automated staining instrument.

### 4.3. CTC Enrichment

Blood samples were processed within 4 h of collection as previously described [29,30]. In brief, whole bloods were collected in K2EDTA tubes (BD-Plymouth, Plymouth, UK) and underwent red blood cell lysis (Astral Scientific, Taren Point, Australia) in a 1 part blood to 3 parts lysis buffer to reduce the cellular component passing through the microfluidic chip. After RBC lysis, the blood sample was centrifuged at 300× *g* for 15 min. The cellular pellet was resuspended in 1X PBS (Thermo Scientific, Waltham, MA, USA) and passed through the spiral chip using a syringe pump (Chemyx, Stafford, TX, USA). After two rounds of enrichment to reduce the background leukocyte contamination, the final CTC output was collected and spun down at 300× *g* for 5 min (Shandon Cytospin, Thermofisher Scientific).

### 4.4. CTC Immunophenotyping

The CTC output was transferred onto poly-l-lysine-coated glass slides (Sigma-Aldrich, St. Louis, MO, USA) for phenotyping by immunocytochemistry. Cells were stained using the CellSearch^®^ antibody cocktail which targets pan-Cytokeratin, CD45, and DAPI (Menarini-Silicon Biosystems, Bologna, Italy). Slides were incubated with the staining reagents as previously described [14,15]. The slides were coverslipped and imaged on the Zeiss Axio Z2 microscope (Carl Zeiss, Ontario, CA, USA). Preliminary results were categorized into CTC-positive or CTC-negative events prior to performing DNA FISH.

### 4.5. DNA Fluorescence In Situ Hybridization

The immunofluorescence signal was removed by incubating the slides at 50 °C in a stripping buffer (2% SDS, 0.8% β-mercaptoethanol, 0.0625 M Tris-HCl, pH 6.8) for approximately 15 min with agitation. The slides were then washed in 1X PBS three times for 5 min. The slides were then fixed in 4% paraformaldehyde (Thermofisher Scientific, USA) and dehydrated via an ethanol series (70%, 85%, and 96%). Slides were treated with RNase (4 mg/mL) (Sigma, USA) and fluorescence in situ hybridization (FISH) carried out using the Vysis LSI ALK break-apart probes (Abbott, USA), coverslipped and sealed with rubber cement, placed in a humid chamber at 37 °C overnight (18–20 h), and counterstained with DAPI as described by the manufacturer. To preserve the signal, prolong gold antifade medium (Invitrogen, USA) was applied prior to coverslipping and imaging. Slides were imaged on the Zeiss Axio Imager Z2 microscope. ALK-positive (ProbeChek 06N38-010) and ALK-negative (ProbeChek 06N38-005) control tissue slides were sourced from Abbott Molecular, USA.

### 4.6. 3D DNA FISH

Slides were imaged on the Zeiss Axio Z2 microscopy at 63X under oil immersion. FISH scanning parameters (z stack, distance between z-stacks, and exposure times) were optimized for FISH signal identification. A multiexposure protocol was developed for optimal capture of signal using a range of between 5 and 25 stacks, and a distance of 0.1–0.5 µm between two successive stacks. Zen software (Zeiss) was used to interrogate the 3D images of the CTCs. Signals were captured by experienced users and the ALK status validated by an experienced molecular pathologist. In native ALK cells, overlapping of the 3′ and 5′ signals produced a fusion signal, which was yellow. The characteristic ALK translocation was observed when there was a split of the 3′ and 5′ signal of more than two signal diameters. The number of ALK-rearranged nuclei per cytospot was enumerated and reported as a percentage from the total number of CTCs identified.

## 5. Conclusions

In summary, we found that imaging CTCs for molecular alterations using 3D DNA FISH for ALK translocations was a feasible method for analyzing CTCs in NSCLC patients. As part of a larger study, we will focus on serial sampling of these patients to understand the changes in CTCs over the course of therapy.

## Supplementary Materials

The following are available online at https://www.mdpi.com/2073-4409/9/6/1465/s1, Video. ALK-translocated CTCs imaged through the nuclear volume to show the separation of ALK signal within the multiple planes (imaged and analyzed on the Nikon Axio Z2 microscope (Carl Zeiss)).

## References

[1] Noone A.M., Howlander N., Krapcho M., Miller D., Brest A., Yu M., Ruhl J., Tatalovich Z., Mariotto A., Lewis D.R. (2018). SEER Cancer Statistics Review. https://seer.cancer.gov/archive/csr/1975_2015/.

[2] Camidge D.R., Kono S.A., Flacco A., Tan A.-C., Doebele R.C., Zhou Q., Crino L., Franklin W.A., Varella-Garcia M. (2010). Optimizing the detection of lung cancer patients harboring anaplastic lymphoma kinase (ALK) gene rearrangements potentially suitable for ALK inhibitor treatment. Clin. Cancer Res..

[3] Minca E.C., Portier B.P., Wang Z., Lanigan C., Farver C.F., Feng Y., Ma P.C., Arrossi V.A., Pennell N.A., Tubbs R.R. (2013). ALK status testing in non-small cell lung carcinoma: Correlation between ultrasensitive IHC and FISH. J. Mol. Diagn. JMD.

[4] Shaw A.T., Solomon B. (2011). Targeting anaplastic lymphoma kinase in lung cancer. Clin. Cancer Res..

[5] Choi Y.L., Soda M., Yamashita Y., Ueno T., Takashima J., Nakajima T., Yatabe Y., Takeuchi K., Hamada T., Haruta H. (2010). EML4-ALK mutations in lung cancer that confer resistance to ALK inhibitors. N. Engl. J. Med..

[6] Abbosh C., Birkbak N.J., Wilson G.A., Jamal-Hanjani M., Constantin T., Salari R., Le Quesne J., Moore D.A., Veeriah S., Rosenthal R. (2017). Phylogenetic ctDNA analysis depicts early-stage lung cancer evolution. Nature.

[7] Revelo A.E., Martin A., Velasquez R., Kulandaisamy P.C., Bustamante J., Keshishyan S., Otterson G. (2019). Liquid biopsy for lung cancers: An update on recent developments. Ann. Transl. Med..

[8] Chemi F., Rothwell D.G., McGranahan N., Gulati S., Abbosh C., Pearce S.P., Zhou C., Wilson G.A., Jamal-Hanjani M., Birkbak N. (2019). Pulmonary venous circulating tumor cell dissemination before tumor resection and disease relapse. Nat. Med..

[9] Dive C., Brady G. (2017). SnapShot: Circulating Tumor Cells. Cell.

[10] Kapeleris J., Kulasinghe A., Warkiani M.E., Vela I., Kenny L., O’Byrne K., Punyadeera C. (2018). The Prognostic Role of Circulating Tumor Cells (CTCs) in Lung Cancer. Front. Oncol..

[11] Zhou J., Kulasinghe A., Bogseth A., O’Byrne K., Punyadeera C., Papautsky I. (2019). Isolation of circulating tumor cells in non-small-cell-lung-cancer patients using a multi-flow microfluidic channel. Microsyst. Nanoeng..

[12] Pailler E., Adam J., Barthelemy A., Oulhen M., Auger N., Valent A., Borget I., Planchard D., Taylor M., Andre F. (2013). Detection of circulating tumor cells harboring a unique ALK rearrangement in ALK-positive non-small-cell lung cancer. J. Clin. Oncol. Off. J. Am. Soc. Clin. Oncol..

[13] Pailler E., Faugeroux V., Oulhen M., Catelain C., Farace F. (2017). Routine clinical use of circulating tumor cells for diagnosis of mutations and chromosomal rearrangements in non-small cell lung cancer—ready for prime-time?. Transl. Lung Cancer Res..

[14] Kulasinghe A., Kapeleris J., Kimberley R., Mattarollo S.R., Thompson E.W., Thiery J.P., Kenny L., O’Byrne K., Punyadeera C. (2018). The prognostic significance of circulating tumor cells in head and neck and non-small-cell lung cancer. Cancer Med..

[15] Kulasinghe A., Kapeleris J., Cooper C., Warkiani M.E., O’Byrne K., Punyadeera C. (2019). Phenotypic Characterization of Circulating Lung Cancer Cells for Clinically Actionable Targets. Cancers.

[16] Tan C.L., Lim T.H., Lim T., Tan D.S., Chua Y.W., Ang M.K., Pang B., Lim C.T., Takano A., Lim A.S. (2016). Concordance of anaplastic lymphoma kinase (ALK) gene rearrangements between circulating tumor cells and tumor in non-small cell lung cancer. Oncotarget.

[17] Provencio M., Perez-Callejo D., Torrente M., Martin P., Calvo V., Gutierrez L., Franco F., Coronado M.J., Cruz-Bermudez J.L., Ruiz-Valdepenas A.M. (2017). Concordance between circulating tumor cells and clinical status during follow-up in anaplastic lymphoma kinase (ALK) non-small-cell lung cancer patients. Oncotarget.

[18] Lin P.P. (2018). Aneuploid CTC and CEC. Diagnostics.

[19] Gué M., Messaoudi C., Sun J.S., Boudier T. (2005). Smart 3D-fish: Automation of distance analysis in nuclei of interphase cells by image processing. Cytom. Part A J. Int. Soc. Anal. Cytol..

[20] Bolland D.J., King M.R., Reik W., Corcoran A.E., Krueger C. (2013). Robust 3D DNA FISH using directly labeled probes. J. Vis. Exp..

[21] Khoo B.L., Warkiani M.E., Tan D.S., Bhagat A.A., Irwin D., Lau D.P., Lim A.S., Lim K.H., Krisna S.S., Lim W.T. (2014). Clinical validation of an ultra high-throughput spiral microfluidics for the detection and enrichment of viable circulating tumor cells. PLoS ONE.

[22] Ilie M., Long E., Butori C., Hofman V., Coelle C., Mauro V., Zahaf K., Marquette C.H., Mouroux J., Paterlini-Brechot P. (2012). ALK-gene rearrangement: A comparative analysis on circulating tumour cells and tumour tissue from patients with lung adenocarcinoma. Ann. Oncol. Off. J. Eur. Soc. Med Oncol..

[23] Dong J., Zhu D., Tang X., Qiu X., Lu D., Li B., Lin D., Zhou Q. (2019). Detection of Circulating Tumor Cell Molecular Subtype in Pulmonary Vein Predicting Prognosis of Stage I-III Non-small Cell Lung Cancer Patients. Front. Oncol..

[24] Pailler E., Faugeroux V., Oulhen M., Mezquita L., Laporte M., Honore A., Lecluse Y., Queffelec P., NgoCamus M., Nicotra C. (2019). Acquired Resistance Mutations to ALK Inhibitors Identified by Single Circulating Tumor Cell Sequencing in ALK-Rearranged Non-Small-Cell Lung Cancer. Clin. Cancer Res..

[25] Punnoose E.A., Atwal S., Liu W., Raja R., Fine B.M., Hughes B.G., Hicks R.J., Hampton G.M., Amler L.C., Pirzkall A. (2012). Evaluation of circulating tumor cells and circulating tumor DNA in non-small cell lung cancer: Association with clinical endpoints in a phase II clinical trial of pertuzumab and erlotinib. Clin. Cancer Res..

[26] McDaniel A.S., Ferraldeschi R., Krupa R., Landers M., Graf R., Louw J., Jendrisak A., Bales N., Marrinucci D., Zafeiriou Z. (2017). Phenotypic diversity of circulating tumour cells in patients with metastatic castration-resistant prostate cancer. BJU Int..

[27] Catelain C., Pailler E., Oulhen M., Faugeroux V., Pommier A.-L., Farace F., Magbanua M.J.M., Park J.W. (2017). Detection of Gene Rearrangements in Circulating Tumor Cells: Examples of ALK-, ROS1-, RET-Rearrangements in Non-Small-Cell Lung Cancer and ERG-Rearrangements in Prostate Cancer. Isolation and Molecular Characterization of Circulating Tumor Cells.

[28] Aya-Bonilla C.A., Marsavela G., Freeman J.B., Lomma C., Frank M.H., Khattak M.A., Meniawy T.M., Millward M., Warkiani M.E., Gray E.S. (2017). Isolation and detection of circulating tumour cells from metastatic melanoma patients using a slanted spiral microfluidic device. Oncotarget.

[29] Kulasinghe A., Schmidt H., Perry C., Whitfield B., Kenny L., Nelson C., Warkiani M.E., Punyadeera C. (2018). A Collective Route to Head and Neck Cancer Metastasis. Sci. Rep..

[30] Kulasinghe A., Tran T.H., Blick T., O’Byrne K., Thompson E.W., Warkiani M.E., Nelson C., Kenny L., Punyadeera C. (2017). Enrichment of circulating head and neck tumour cells using spiral microfluidic technology. Sci. Rep..

[31] Warkiani M.E., Khoo B.L., Wu L., Tay A.K.P., Bhagat A.A.S., Han J., Lim C.T. (2016). Ultra-fast, label-free isolation of circulating tumor cells from blood using spiral microfluidics. Nat. Protoc..

