# Supplementary figures and images for: The Use of Three-Dimensional DNA Fluorescent In Situ Hybridization (3D DNA FISH) for the Detection of Anaplastic Lymphoma Kinase (ALK) in Non-Small Cell Lung Cancer (NSCLC) Circulating Tumor Cells
Source: Cells. 2020 Jun 15;9(6):1465. doi: 10.3390/cells9061465 (PMC7349512; doi:10.3390/cells9061465)

## Slide 1
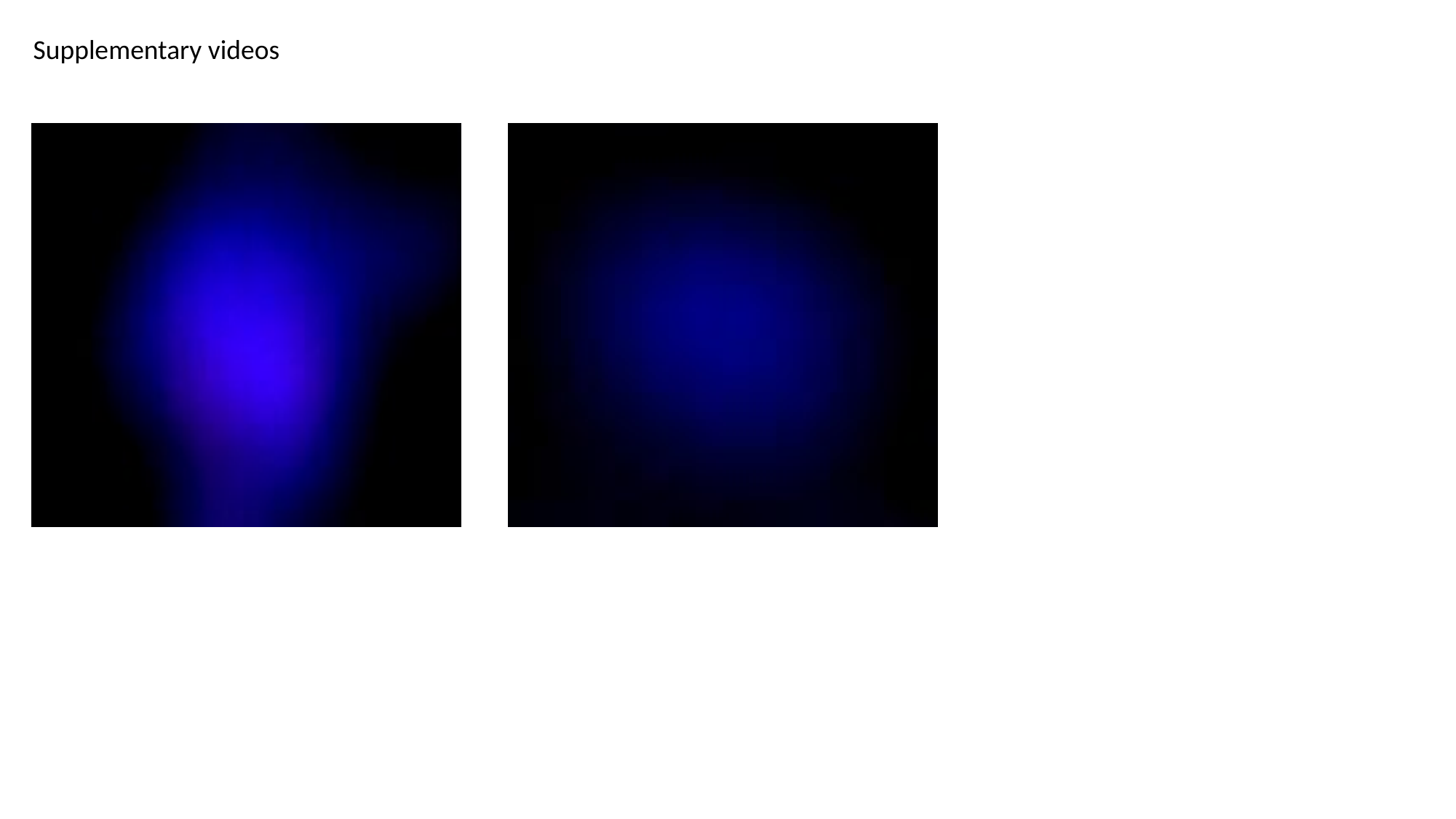

Supplementary videos

Supplement: Supplementary file 1 [file cells-09-01465-s001.zip › cells-811394-SI/supplemtary video 1..pptx]
